# Context specificity of latent inhibition in the snail *Cornu aspersum*

**DOI:** 10.1007/s10071-022-01632-6

**Published:** 2022-05-17

**Authors:** Judit Muñiz-Moreno, Ignacio Loy

**Affiliations:** Department of Psychology, University of Oviedo, Plaza de Feijoo s/n, 33003 Oviedo, Spain

**Keywords:** Invertebrate learning, Pavlovian conditioning, Photoperiod, Light context

## Abstract

The present study was conducted to assess the context specificity of latent inhibition (LI) in the snail *Cornu aspersum*, using the appetitive Pavlovian Conditioning procedure of tentacle lowering. Snails experienced an odorous conditioned stimulus (CS) without any consequence before being conditioned with food. The conditioned stimulus preexposure occurred in the same context than the conditioning and the test context or in the different context. The study was performed in two replicas in which the photoperiod was defined by level of illumination and time of day (circadian replica) or was defined only by light (light replica). Both replicas showed that the CS preexposure in the same context as conditioning produced a delay in the acquisition of the conditioned response (CR). However, when the CS preexposure took place in a different context than the conditioning context, an equivalent level of CR as that observed in controls without preexposition to CS was shown. These results are congruent with context specificity of LI and they provide the first evidence of this phenomenon in terrestrial mollusks. Learning processes and theories involved in this phenomenon are also debated in the paper.

## Introduction

In appetitive Pavlovian Conditioning, a CS becomes associated with the taste and/or the nutritive properties of an Unconditioned Stimulus (US), usually food. It is possible to modulate the strength of this association by manipulating the variables effective in standard conditioning paradigms. One of the most relevant variables is the experience with the CS previous to conditioning. When the conditioned stimulus (CS) is repeatedly exposed to a neutral stimulus subsequent conditioning is retarded when that stimulus is used as a CS (Lubow and Weiner 2010). This phenomenon, named Latent Inhibition (LI), has been explained by two basic approaches: one is based on failure in the CS-US acquisition (Acquisition models) and the other on the CS-US association retrieval (Retrieval models). In addition, as with several other learning phenomena, LI shows contextual specificity (Hall and Honey 1989) and this effect is predicted by both theoretical accounts.

The effect of context on learning phenomena has been studied mainly in vertebrate animals, for example: habituation (e.g., Siegel 1977; see Dissegna et al. 2021 for a review), negative transfer (e.g., Swartzentruber and Bouton 1986), renewal (e.g., Bernal-Gamboa et al. 2012; Bouton and Bolles 1979; Mesich et al. 2021) or overshadowing (Kwok and Boakes 2017). However, very little has been said in the literature about this issue in invertebrate species (Howard et al. 2017, perception of contextual size illusions in honey bees; McComb et al. 2002, renewal in *Lymnaea stagnalis*; Loy et al. 2020, renewal in terrestrial snails; Hermitte et al. 1999; Predreira et al. 1995, 1996; Pereyra et al. 2000; Tomsic et al. 1998, context specificity of habituation in crabs; Reyes-Jiménez et al. 2020, 2021, effect of the context specificity of habituation in earth worms; Lau et al. 2013; Rankin 2000, effect of the context specificity of habituation in *C. elegans*; see Dissegna et al. 2021 for a review).

Specifically, much evidence has been reported in vertebrate species in the study of LI, including humans (e.g., Ginton et al. 1975; Lubow and Moore 1959; Silver 1973; Zalstein-Orda and Lubow 1995), rodents (e.g., Hall and Pearce 1979; Kiernan and Westbrook 1993; Lubow et al. 1968; Reiss and Wagner 1972), fishes (e.g., Ferrari and Chivers 2006; Mitchell et al. 2011; Shishimi 1985) or amphibians (Daneri and Muzio 2015; Ferrari and Chivers 2009, 2011; Gonzalo et al. 2013). Moreover, against Lubow and Weiner’s (2010) claim that the hippocampus is essential for LI to occur, this phenomenon has also been observed in invertebrate species with a simpler nervous system, such as crustaceans (e.g., Acquistapace et al. 2003) insects (e.g., Abramson et al. 2005; Abramson and Bitterman 1986; Bennett et al. 2021; Bitterman et al. 1983; Chandra et al. 2000, 2001, 2010; Cook et al. 2019; Fernández et al. 2012; Jacob et al. 2021; Petersen 2017) or gastropods (e.g., Escobar et al. 2014; Loy et al. 2006).

A considerable amount of literature has been published on context specificity of LI in vertebrates (e.g., Archer et al. 1986; Hall and Channell 1985; Hall and Honey 1989; Lovibond et al. 1984; Miller et al. 2015; Miguez et al. 2018; Molero-Chamizo 2018; Westbrook et al. 2000). However, the only evidence of context specificity of LI in invertebrates has recently been found by Jacob et al. (2021) in *Drosophila melanogaster*. In this study, it was shown that flies preexposed to the CS in a different context than conditioning and the test context reached equivalent levels of CR as flies which were preexposed to a different stimulus than the CS (Jacob et al. 2021).

The aim of the present paper is to study the context specificity of LI in the snail *Cornu aspersum*. The tentacle lowering procedure was employed because it is a robust appetitive Pavlovian preparation and has been used in several studies such as simple conditioning (Ungless 1998, 2001); LI, overshadowing, second-order conditioning and sensory preconditioning (Loy et al. 2006); conditioned inhibition (Acebes et al. 2009); blocking (Acebes et al. 2012; Prados et al. 2013); spontaneous recovery and reinstatement (Álvarez et al. 2014); and renewal (Loy et al. 2020). According to the stimulus employed as context, the work reported here was made in two different replicas. In the first one, the circadian replica, the context used was photoperiod (defined by the hour of the day and the lighting) as found in the study of Loy et al. (2020). In the second one, the light replica, only light was used as context. The second replica was carried out to find out whether the same results as the circadian replica should be observed with a simpler procedure, as the element “hour of the day” is eliminated and the experiment is carried out in fewer hours.

## Method

The present study was designed to determine the context specificity of LI in snail *Cornu aspersum.* Subjects were divided in four groups according to the stimulus preexposure and the preexposure context. Moreover, two replicas were created on the basis of context stimulus: photoperiod (the circadian replica) and light (the light replica). In both replicas, it was expected that subjects which were preexposed to the CS_1_ in the same context as conditioning would show a delay in the conditioning acquisition, congruent with LI. In addition, subjects preexposed to the CS_1_ in a different context than the conditioning context were expected to show the acquisition of the CS-US association, congruent with context specificity of LI.

### Subjects and housing

The subjects used in this study were the common snails *Cornu aspersum*, which were collected from the wild in a garden from the small town of Noreña (Asturias). They lived grouped among the garden stones and their food was the green leaves of the ferns present in their habitat. They were manually collected from their habitat and taken directly to the laboratory, where they were maintained and prepared for each experimental replica.

52 adult snails were employed, with a mean shell diameter of 25.94 mm (range 20–32 mm) for the circadian replica, whereas 53 adult snails with a mean shell diameter of 29.06 mm (range 22–33 mm) were used for the light replica. Snails were individually housed in plastic cages (50 × 50 × 100 mm) with air holes. The house boxes were placed in a room with a constant temperature of 22 ºC and a reversed 12 light/dark cycle, starting at 06:00 am. They were given access to a small amount of water and ad libitum food, which was composed of corn grains for poultry, and prior to the start of the experiment, they were food-deprived for 10 days. At the end of the experiment, snails were given food ad libitum (corn grains) and placed back into the wild, but in a different garden, 50 km away from the place where they were collected to avoid their recapture.

### Apparatus and stimuli

The experimental set was a plastic perforated surface (390 × 360 mm; 5.5 mm diameter holes, roughly 2 mm apart from one to another) placed 65 mm above the surface of a table and the experimental room was maintained at 22 ºC. The context stimuli were two types of lights to reproduce the light/dark context in both replicates of the experiment. A white light (LED 5.5 W) was used as the light context, whereas a red light (LED 3 W) was used as the dark context, given that prior research established the snail’s spectral sensitivity range in 390–580 nm (Barker 2006), which is lower than the red light range (620–750 nm), so the red light cannot be perceived by snails. By contrast, the red light is perceived by humans and the use of this light allows us to observe the response of the subject properly. Also, two solutions, one obtained from mango and another one from coconut (oil brand *La Casa de Los Aromas*, 2 ml/L of distilled water) were used as the CSs, and carrot was used as the US. The pieces of carrot had a mean diameter of 27 mm (range 22–29 mm) and were 1 mm thick.

### Procedure

In this experiment, tentacle lowering was measured as the CR by one observer, who was not aware of the group to which each subject belonged. This measure consisted of counting in real time the number of times the left tentacle descended below an imaginary line, drawn horizontally just above the head of the snail (Ungless 1998, 2001).

Snails were randomly divided into four groups based on the context in which subjects received the preexposure and the kind of stimulus presented during the preexposure. Subjects from *same context-preexposure* group were preexposed to the odour used during conditioning (CS_1_) in the same context as the conditioning and the tests. On the contrary, subjects from *different context-preexposure* group were preexposed to the CS_1,_ but in a different context than the conditioning and the tests. From *same context-no preexposure* group, subjects were preexposed to a different odour from that used during conditioning (CS_2_) in the same context as the conditioning and the tests. Finally, subjects from *different context-no preexposure* group were preexposed to the CS_2_ in the different context. The role of the odours was not counterbalanced, so the odour of mango was used as CS_1_ and the odour of coconut was used as CS_2_.

In addition, this experiment was carried in two replicas: the circadian replica, in which the photoperiod was used as context, determined by the hour of the day and the illumination level; and the light replica, in which only light was used as context. In both replicas, the context was counterbalanced so, for half of the subjects in each group, the conditioning and the tests were made in the light context and for the other half, they were made in the dark context. The circadian replica was started at 8:00 a.m. and 8:00 p.m. and finished at 13:00 p.m. and 01:00 a.m., respectively, and the light replica started at 8:00 a.m. and finished at 13:00 p.m. At the beginning of each trial, snails were sprayed with fresh water to induce their activity and at the end of each trial they were returned to their home boxes without any access to the stimuli used throughout the experimental phases.

i. *Pre-Test*
  In this phase, the tentacle lowering response was measured for each subject individually. Based on the group to which they belonged, snails were exposed to CS_1_ or CS_2_ for 2 min. This odour was placed below the perforated surface in a dish containing four cotton pads and each one was impregnated with 2 ml of the solution.
ii. *Preexposure*
  During the preexposure phase, the odour (CS_1_ or CS_2_) was presented for 2 min. The odour was placed in the same way as pre-test and 6 trials were made during the day with an intertrial interval (ITI) of 58 min.
iii. *Conditioning and Test*

**Fig. 1:**
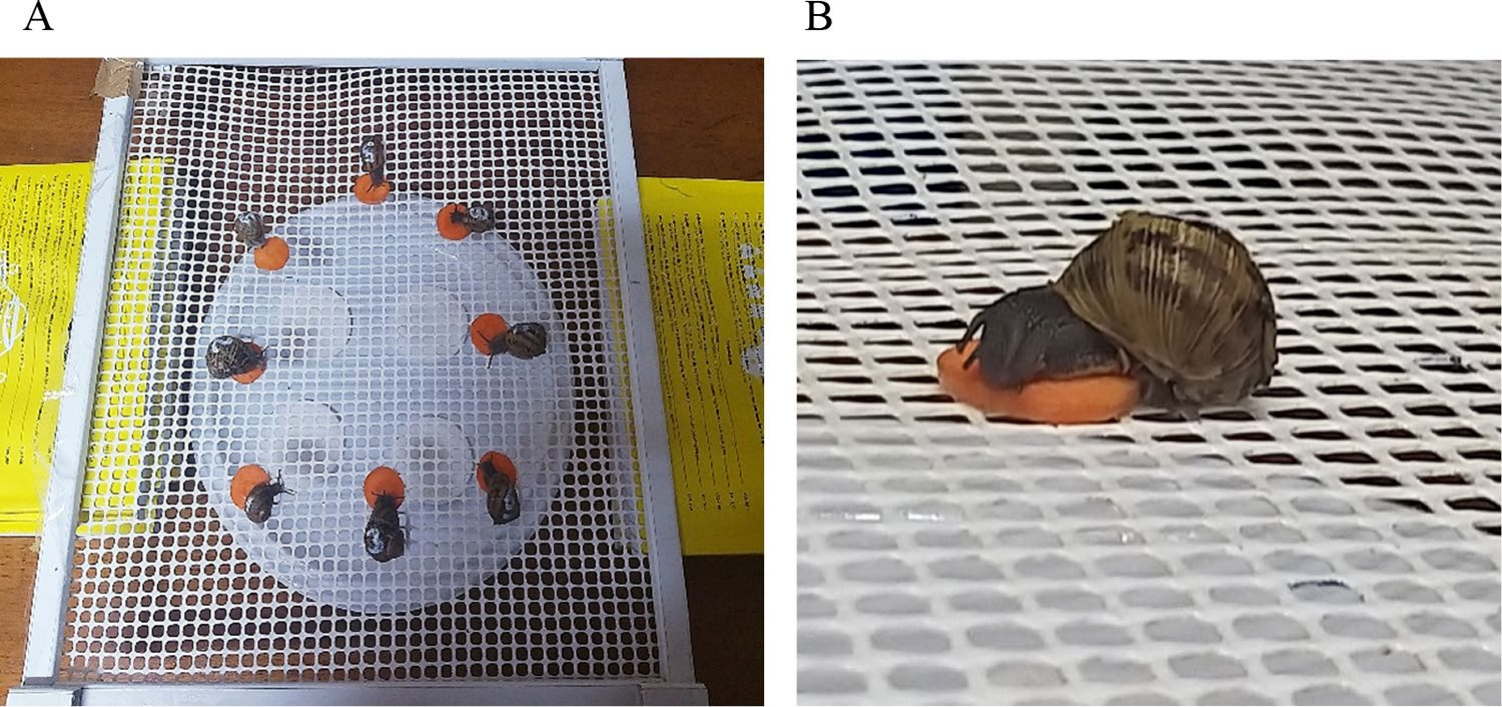
Conditioning Phase. Panel **A** shows the experimental setting and how conditioning was performed in groups. Panel **B** shows a snail eating during conditioning

In the conditioning phase, all the groups were exposed to CS_1_ paired with access to food (US) for 2 min. A piece of carrot was placed in front of snail whereas the odour was placed in the same way as in the previous phases. 3 trials were performed during the day with an ITI of 58 min (see Fig. 1).

On a different day than the conditioning phase, the test was carried out in the same way as the pre-test, using the same context as conditioning. The conditioning-test cycle was made 3 times, so conditioning was repeated 9 times and the test 3 times. The experimental design is summarized in Table 1.

**Table 1:** Experimental design for both replicas

Groups	Pre-Test (Day 1)	Preexposure (Day 2)	Conditioning (Days 3–5-7)	Test (Days 4–6-8)
Same context preexposure	(Sa) CS_1_	(Sa) CS_1_		
Different context preexposure	(Di) CS_1_	(Di) CS_1_	(Sa) CS_1_ + US	(Sa) CS_1_
Same context no preexposure	(Sa) CS_2_	(Sa) CS_2_		
Different context no preexposure	(Di) CS_2_	(Di) CS_2_		

^*^ CS_1_ was a mango solution, CS_2_ was a coconut solution, US was a piece of carrot. The abbreviation Sa indicates “same context” used throughout all the experimental treatment and the abbreviation “Di” indicates the different context (the contextual cue was the photoperiod in the circadian replica and light was the contextual cue in the light replica). Also, the light and the dark contexts were counterbalanced, so for half of the subjects Sa was the dark context and Di was the light context and for the other half Sa was the light context and Di was the dark context. The symbol “ + ” indicates that stimuli were presented simultaneously

### Statistical analysis

**Fig. 2:**
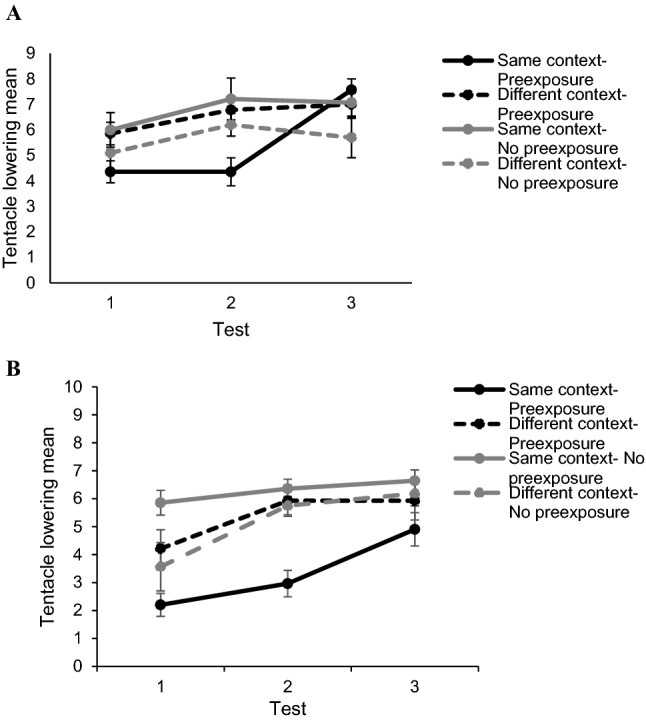
Experimental Results. This figure represents the mean number of tentacle-lowering responses (CR) made by the different groups: same context-preexposure, different context-preexposure, same context-no preexposure and different context-no preexposure throughout the three experimental tests for the circadian variant (panel **A**) and the light variant (panel **B**). Vertical bars represent SEMs

The number of times that subjects lowered the left tentacle during the pre-tests and tests was measured. One-way analysis of variance was used in the pre-test analysis. Also, the repeated-measures ANOVAs were carried out to observe if there were any differences in the counterbalanced training context and to analyse the main results shown in Fig. 2. Finally, the differences among the groups observed in test 2 were compared using the Univariate ANOVA and the Bonferroni pairwise comparisons. These analyses were taken into account for each replica of the study. In addition, the level of significance used was α = 0.05 and the effect sizes for ANOVAs were reported as partial Eta-square (η^2^_p_). Data management and analysis was performed using SPSS v21 (SPSS Inc., Chicago, IL, USA).

## Results

Figure 2 shows the tentacle lowering mean for each group in the three conditioning tests. Panel A presents the results of the circadian replica, whereas Panel B provides the results of the light replica. As can be seen from Panel A, all groups showed an equivalent level of conditioning throughout the three tests except for the group *same context-preexposure*. This group presented a lower conditioning level than the rest of the groups in the first two tests, this difference being higher in test 2. Nevertheless, CR for the *same context-preexposure* group increased during test 3, showing an equivalent level of conditioning to the other groups. This description was corroborated by the statistical analyses. The same effect was observed in Panel B, but during test 1 the CR of the of *same context*-*no preexpsoure* group was higher than the CR for the rest of the groups. However, this difference was not significant as the statistical analyses show.

The first set of analyses examined the effect of context counterbalancing to see if there were any significant differences in the CR between-subjects which received the conditioning and the tests in the light context and the subjects which received the conditioning and the tests in the dark context. A repeated-measures ANOVA was carried out with the pre-test and the tests as the within-subjects factor and the preexposure context (if the preexposure was made in the same context as conditioning or in the different context), the stimulus preexposure (the CS_1_ preexposure or CS_2_ preexposure) and the training context (if the conditioning and the tests were performed in the light or in the dark context) as the between-subjects factors.

In the circadian replica, the effect of the training context was significant [ANOVA: *F*_1, 44_ = 6.786, *P* = 0.012, η^2^_p_ = 0.134], but there were no significant interactions between the training context and the other factors, neither with the stimulus preexposure [ANOVA: *F*_1, 44_ = 0.710, *P* = 0.404, η^2^_p_ = 0.016] nor with the preexposure context [ANOVA: *F*_1, 44_ = 1.721, *P* = 0.196, η^2^_p_ = 0.038]. Also, the second-degree interaction was not significant [ANOVA: *F*_1, 44_ = 0.676, *P* = 0.415, η^2^_p_ = 0.015]. These results reflect higher means in one context than in the other but, since they are counterbalanced, the effect was offset and it did not affect the validity of the results. Thus, the data were collapsed.

In the light replica, the analyses did not show a significant effect of the training context [ANOVA: *F*_1, 45_ = 0.336, *P* = 0.565, η^2^_p_ = 0.007]. Also, there were no significant differences in the interaction of the training context with the stimulus preexposure [ANOVA: *F*_1, 45_ = 0.926*, P* = 0.341, η^2^_p_ = 0.020], the interaction with the preexposure context [ANOVA: *F*_1, 45_ = 0.098, *P* = 0.755, η^2^_p_ = 0.002] and in the second-degree interaction [ANOVA: *F*_1, 45_ = 0.015, *P* = 0.902, η^2^_p_ = 0.000]. As in the circadian replica, these data were collapsed.

The second set of analyses was made to find out whether there was a preference for one of the odours during the pre-test for each experimental replica. One-factor ANOVA was made with the pre-test as dependent variable and the four groups as independent variable. In both replicas, there were no significant differences in the preference for one odour: the circadian replica [ANOVA: *F*_3, 48_ = 1.004, *P* = 0.399, η^2^_p_ = 0.059] and the light replica [ANOVA: *F*_3, 49=_ 1.439, *P* = 0.243, η^2^_p_ = 0.081].

Then, several analyses were performed to examine the data represented in Fig. 2. For each replica, a repeated-measures ANOVA was carried out with the tests as the within-subjects factor, whereas the preexposure context and the stimulus preexposure were the between-subjects factors.

In the circadian replica, the analysis indicated a significant effect of the tests [ANOVA: *F*_2, 96_ = 10.743, *P* < 0.001, η^2^_p_ = 0.183], but not of the stimulus preexposure [ANOVA: *F*_1, 48_ = 0.247, *P* = 0.621, η^2^_p_ = 0.005] or of the preexposure context [ANOVA: *F*_1, 48_ = 0.001, *P* = 0.979, η^2^_p_ = 0.000]. Moreover, it showed a significant effect of the interactions between the tests and the stimulus preexposure [ANOVA: *F*_2, 96_ = 5.057, *P* = 0.009, η^2^_p_ = 0.095], the tests and the preexposure context [ANOVA: *F*_2, 96_ = 3.619, *P* = 0.032, η^2^_p_ = 0.070], and the preexposure context with the stimulus preexposure [ANOVA: *F*_1, 48_ = 5.928, *P* = 0.019, η^2^_p_ = 0.110]. Nevertheless, the second-degree interaction was not significant [ANOVA: *F*_2, 96_ = 2.091, *P* = 0.129, η^2^_p_ = 0.042].

For the light replica, the statistical analyses presented a significant effect of the tests [ANOVA: *F*_2, 98_ = 20.667, *P* < 0.001, η^2^_p_ = 0.297] and the stimulus preexposure [ANOVA: *F*_1, 49_ = 9.771, *P* = 0.003, η^2^_p_ = 0.166], but not a significant effect of the preexposure context [ANOVA: *F*_1, 49_ = 0.748, *P* = 0.391, η^2^_p_ = 0.015]. In addition, the analyses of the interactions showed a significant effect between the preexposure context and the stimulus preexposure [ANOVA: *F*_1, 49_ = 16.508, *P* < 0.001, η^2^_p_ = 0.252] and the preexposure context with the tests [ANOVA: *F*_2, 98_ = 3.119, *P* = 0.049, η^2^_p_ = 0.060], but not for the interaction between the preexposure and the tests [ANOVA: *F*_2, 98_ = 0.850, *P* = 0.410, η^2^_p_ = 0.017] or for the second-degree interaction [ANOVA: *F*_2, 98_ = 2.393, *P* = 0.108, η^2^_p_ = 0.047].

The results for both experimental replicas suggested that subjects showed different CR levels depending on the stimulus preexposed (CS_1_ or CS_2_) and the context of the preexposure phase (light or dark context). According to Fig. 2, in both panels (A and B), which represent the circadian and the light replicas, respectively, the main differences among the groups were observed in test 2. So, an analysis of the effect of the test was carried out with the Bonferroni pairwise comparisons to corroborate this issue.

In both replicas, it was shown that there existed significant differences between test 1 and test 2 (circadian replica: test1 MDS 5.34 ± 0.28, test2 MDS 6.13 ± 0.34, *P* = 0.026; light replica test1 MDS 3.96 ± 0.35, test2 MDS 5.26 ± 0.28, *P* < 0.001) and test 1 and test 3 (circadian replica test1 MDS 5.34 ± 0.28, test3 MDS 6.92 ± 0.28, *P* < 0.001; light replica test1 MDS 3.96 ± 0.35, test3 MDS 5.83 ± 0.28, *P* < 0.001). Nevertheless, there were no significant differences between test 2 and test 3 (circadian replica test2 MDS 6.13 ± 0.34, test3 MDS 6.92 ± 0.28, *P* = 0.117; light replica test2 MDS 5.26 ± 0.28, test3 MDS 5.83 ± 0.28, *P* = 0.055).

These results supported the idea that the main differences among the groups take place in test 2. The results shown in test 2 were analysed with a Univariate ANOVA for each replica. The context preexposure and the stimulus preexposure were the between-subjects factors.

The analysis of test 2 in the circadian replica did not show a significant effect of the preexposure context [ANOVA: *F*_1, 48_ = 1.234, *P* = 0.272, η^2^_p_ = 0.025] or the stimulus preexposure [ANOVA: *F*_1, 48_ = 3.183, *P* = 0.081, η^2^_p_ = 0.062]. However, it showed significant differences in the interaction between them [ANOVA: *F*_1, 48_ = 7.314, *P* = 0.009, η^2^_p_ = 0.132].

In the light replica, a significant effect of the preexposure context was revealed [ANOVA: *F*_1, 49_ = 7.304, *P* = 0.009, η^2^_p_ = 0.130] as well as in the stimulus preexposure [ANOVA: *F*_1, 49_ = 13.457, *P* < 0.001, η^2^_p_ = 0.215]. Furthermore, as in the circadian replica, the effect of the interaction between them was significant [ANOVA: *F*_1, 49_ = 16.571, *P* < 0.001, η^2^_p_ = 0.253].

In test 2, both replicas showed a significant effect of the interaction between the preexposure context and the stimulus preexposure. This interaction was analysed with the Bonferroni pairwise comparisons. It showed significant differences between *same context—preexposure group* and *same context—no preexposure group* (circadian replica: MDS 4.36 ± 0.55, MDS 7.21 ± 0.82 respectively, *P* = 0.002; light replica: MDS 2.96 ± 0.47, MDS 6.36 ± 0.34 respectively, *P* < 0.001) in which the tentacle-lowering mean of *same context—preexposure group* was lower than the tentacle-lowering mean of *same context- no preexposure group*. These results indicated that there were not equivalent CR in the *same context-preexposure groups* with respect to those that received another CS and it is congruent with the LI effect.

In addition, there were significant differences in the tentacle-lowering mean between *same context-preexposure group* and *different context-preexposure group* (circadian replica: MDS 4.36 ± 0.55, 6.79 ± 0.54 respectively, *P* = 0.007; light replica: MDS 2.96 ± 0.47, MDS 5.93 ± 0.56, respectively *P* < 0.001), so the results suggest that there was an effect of the context involved in the LI performance, which is congruent with context specificity of LI.

However, the analyses did not show significant differences (circadian replica *P* = 0.536; light replica *P* = 0.779) between the tentacle-lowering mean of *the different context—preexposure group* and the mean of the *different context—no preexposure group* (circadian replica: MDS 6.79 ± 0.54, MDS 6.2 ± 0.44; light replica: MDS 5.93 ± 0.56, MDS 5.75 ± 0.33, respectively). These results indicate that there were equivalent conditioning levels in the *different context-preexposure groups* with respect to those that received another CS.

Also, there were no significant differences (circadian replica *P* = 0.286; light replica *P* = 0.343) between the *same context-no preexposure* and *different context- no preexposure* (circadian replica: MDS 7.21 ± 0.82, MDS 6.2 ± 0.44; light replica MDS 6.36 ± 0.34, MDS 5.75 ± 0.33, respectively). These results indicate that there were equivalent conditioning levels in both control groups.

According to Fig. 2 and the statistical analyses for both experimental replicas, during test 1 and test 2 subjects which were preexposed to the CS_1_ in the same context as the conditioning and the tests presented a lower CR, in contrast with subjects which were preexposed to the CS_2_ or were preexposed to CS_1_ but in a different context. These differences among the groups were significant in test 2. Finally, the differences disappeared in test 3 as all the groups showed an equivalent CR. The results of these experiments support the idea that subjects from the *group same context- preexposure* exhibited a conditioning acquisition delay, which can be interpreted as LI. Also, subjects from the group *different context-preexposure* showed an attenuation of LI phenomenon produced by a context change (the context specificity of LI). This effect takes place regardless of the replica. Therefore, both context cues (the photoperiod or the light) were equally effective.

## General discussion

The purpose of the current study was to determine the context specificity of LI in the snail *Cornu aspersum*, using the Pavlovian Conditioning of tentacle lowering procedure. The experiment presented here had two replicas: the circadian replica, in which the photoperiod (determined by the hour of the day and the illumination) was used as context; and the light replica, in which only the light was used as context. The second one (the light replica) was performed to reproduce the results observed in the circadian replica and simplify the procedure.

The study has found that subjects which were preexposed to the CS_1_ in the same context as the conditioning and the tests showed the lowest CR mean during the tests 1 and 2. Nevertheless, in test 3, these subjects reached an equivalent conditioning level as the rest of the groups. These results showed a delay of the conditioning acquisition which can be considered an instance of LI phenomenon. The second major finding was that subjects which were preexposed to the CS_1_ in a different context than the conditioning and the tests showed an equivalent level of CR throughout the three tests as subjects which were preexposed to the CS_2_. In addition, during the first two tests, these subjects presented a higher CR level than subjects which were preexposed to the CS_1_ in the same context as the conditioning and the tests. These results support the idea that a context change during the preexposure of the CS_1_ affects LI. The present study is, therefore, the first attested evidence about context specificity of LI in terrestrial mollusks and can be added to the only study we are aware of that found contextual specificity of LI in an invertebrate species (Jacob et al. 2021). The results obtained in both experimental replicas (the circadian replica and the light replica) show that the use of the photoperiod or the light as context produces an equivalent context specificity. Nevertheless, the use of the light as context offers a simpler experimental procedure.

The results of LI and their context specificity observed in these experimental replicas may be explained by several learning theories. On the one hand, the Acquisition models suggest that the CS preexposure reduces the associative strength of this stimulus with the US during conditioning. Therefore, failure in the acquisition of the CS-US association is produced (e.g., Lubow et al. 1976, 1981; Mackintosh 1975; McLaren et al. 1989; Pearce and Hall 1980; Wagner 1978, 1981; see Serra and De la Casa 1989 for a review). Moreover, the Wagner account (1978, 1981) offers an explanation for the context specificity of LI and it shows that, during the CS preexposure, this stimulus is paired with the context and reduces the associative strength of the CS to establish other associations, which causes the failure in the acquisition of the CS-US association. However, if the preexposure is performed in a different context than conditioning, the CS recovers its associative strength during the conditioning phase and the failure in the acquisition of the CS-US association does not occur.

On the other hand, the Retrieval models predict that the CS-US association will be acquired during the conditioning phase, but the CS preexposure will interfere in the performance CS-US association, giving a failure in memory retrieval. In addition, this interference is modulated by the context so, if the CS preexposure takes place in a different context than conditioning context, this interference does not happen and the memory of the CS-US association is recovered (e.g., Bouton 1993; Miller et al. 1986; Escobar and Miller 2010; Schmajuk et al. 1996; Weiner 1990; see Lubow and Weiner 2010 for a review).

According to the literature, several studies about the context effect in learning phenomena suggest that the association between the context and the US is not enough to explain the phenomenon. For example, a conditioned suppression study showed that an association between the context and the US is not necessary for the influence of the context over the CS performance to occur (see Bouton and King 1983, 1986). However, our study was not specifically designed to evaluate which of these models better explains the present results. For example, no test was included to measure the conditioning level of the contexts and no tests in a third context (a neutral context) were included either, which would have allowed us to discern between the Wagner account (1978, 1981) and the Retrieval models.

Prior studies have noted the importance of neural mechanisms involved in the LI performance. For example, in the attentional model of Schmajuk (1987) it is suggested that the hippocampus is involved in several psychological processes such as the inhibition of the response or the retrieval of contextual information which are necessary for LI to occur (Schmajuk 1987). This has been confirmed by extensive research using humans and other vertebrate species, such as rodents (e.g.,Solomon and Moore 1975; Puga et al. 2007; Weiner 1990). Furthermore, it has been shown that other brain areas are involved in LI such as the ventral cochlear nucleus, the perirhinal cortex, the accumbens nucleus, the entorhinal cortex (Puga et al. 2007; Weiner 2003) the mesolimbic system (e.g., Weiner 1990) or the parabrachial nucleus (e.g., Gasalla et al. 2016). However, in recent years, the research on LI has developed considerably, providing relevant evidence for this phenomenon taking place in simple animals without these nervous structures, including the first evidence about context specificity of LI in the insect *Drosophila melanogaster* (Jacob et al. 2021).

One limitation of the present study and other investigations based on Classical Conditioning procedures is that they are susceptible to be confused with the effects of habituation and sensitization, which call in question the LI evidence in invertebrates (Lubow and Weiner 2010). Even though no test has been performed to rule out these alternative explanations for these experiments, a similar procedure (Loy et al.’s (2006) Experiment 1) showed an absence of habituation effects after 6 unreinforced exposures to CS by the unpaired group (Fig. 1A, p. 307). Therefore, it is difficult to explain the results of the present analysis by a phenomenon other than LI.

In addition to this, the studies of LI in invertebrates could reinforce research on neuromodulators which could be present in both vertebrate and in invertebrate animals (e.g., Van Damme et al. 2020). Thus, to understand complex learning phenomena as LI, it is necessary to broaden the range of both learning procedures and subject species.
